# Vitamin D status in patients with knee or hip osteoarthritis in a Mediterranean country

**DOI:** 10.1007/s10195-014-0322-y

**Published:** 2014-10-22

**Authors:** Thomais Goula, Alexandros Kouskoukis, Georgios Drosos, Alexandros-Savvas Tselepis, Athanasios Ververidis, Christos Valkanis, Athanasios Zisimopoulos, Konstantinos Kazakos

**Affiliations:** 1 Department of Orthopaedics, Democritus University of Thrace, University General Hospital of Alexandroupolis, Dragana, Alexandroupolis, 68100 Greece; 2 Department of Nuclear Medicine, Democritus University of Thrace, University General Hospital of Alexandroupolis, Alexandroupolis, Greece; 3 Naval Hospital Of Athens, Athens, Greece

**Keywords:** Vitamin D, Osteoarthritis, Knee osteoarthritis, Hip osteoarthritis

## Abstract

**Background:**

Vitamin D plays an important role in bone mineralization, remodeling, and maintenance and therefore its deficiency may be implicated in the pathogenesis of osteoarthritis (OA). Vitamin D status was evaluated in patients with knee or hip OA scheduled for joint replacement. The impact of anthropometric parameters such as gender, age, and body mass index on vitamin D levels was also examined. The study was conducted in a Mediterranean country (Greece).

**Materials and methods:**

We included 164 patients with knee or hip OA scheduled for joint replacement in this study. Serum levels of 25-hydroxyvitamin D (vitamin D) were measured in routine blood samples taken from the patients at their pre-admission visit, a week before the operation, using radioimmunoassay.

**Results:**

The majority of patients were vitamin D deficient (81.7 %); 15.2 % of them were vitamin D insufficient (hypovitaminosis). Only 3 % of patients were vitamin D sufficient. There was a significantly positive association between vitamin D levels and male gender.

**Conclusion:**

These findings indicate a large percentage of vitamin D deficient patients with knee or hip OA, which is unexpected considering the high annual insolation in northern Greece. Many other possible predisposing factors for OA should be taken into consideration. Whether treatment with vitamin D supplements may provide beneficial effects to these patients and the stage of disease in which this treatment should commence remains an issue for further scientific investigation.

**Level of evidence:**

Level IV.

## Introduction

Vitamin D deficiency is one of the most common and under-diagnosed medical conditions in the world, since a significant proportion of the population in many countries and regions around the world have low vitamin D levels [1–4]. The 25-hydroxyvitamin D level depends on various parameters, including the amount of solar ultraviolet B (UVB) irradiation (determined by the time of day, season [5–7] latitude, skin pigmentation, and use of sunscreen), age [7], dietary habits, gender, obesity [8], and many others [9].

Vitamin D plays an important role in bone mineralization, remodeling, and maintenance and therefore its deficiency may be implicated in the pathogenesis of osteoarthritis (OA) [10, 11]. Although the pathogenesis of OA is still unclear, recent evidence suggests that changes in subchondral bone remodeling—phases of bone absorption and of bone sclerosis––may be responsible for cartilage damage. Vitamin D has been shown to modulate the activity of metalloproteinase enzymes. Low levels of 25(OH)D3 lead to an increased production of degradative enzymes [12]. The theory behind changes in the bone is that low levels of 25-hydroxyvitamin D slow the remodeling response of subarticular bone, resulting in thickening of the subchondral bone, osteophyte formation, and resultant cartilage damage [13].

Prospective epidemiological studies have found an association between dietary intake and serum levels of 25-hydroxyvitamin D and the development or progression of radiographic hip [14, 15] and knee OA [22]. Low serum levels of 25-hydroxyvitamin D have been reported in a significant proportion of patients with OA of hip and knee joints [14, 16–24]. Some authors suggest that achieving vitamin D sufficiency may prevent and/or delay cartilage loss in knee OA [15, 25]. In patients with hip OA who underwent total hip replacement, 25-hydroxyvitamin D levels were found to correlate positively with both pre- and post-operative Harris hip scores. Therefore, it seems that vitamin D deficiency in patients undergoing total hip replacement may be a risk factor for a suboptimal outcome [19].

However, results of other studies do not support an association between the low level of serum 25(OH)D and the development of OA [27–29]. An association of serum 25(OH)D levels with hip or knee OA has therefore not yet been fully established. The authors recommended serum 25(OH)D measurement in any patient with symptoms suggestive of knee OA, particularly at the initial stage of disease [23].

The main purpose of this study was to evaluate the vitamin D status in patients with knee or hip OA scheduled for joint replacement in a Mediterranean country. Associations between vitamin D serum levels and gender, age, and body mass index (BMI) were also investigated.

## Materials and methods

This uncontrolled cohort study was conducted from December 2011 to October 2012 in a Mediterranean country. The study was approved by the hospital’s scientific ethics committee and all patients provided informed consent.

Patients with hip or knee OA scheduled for hip or knee replacement were included in this study. Exclusion criteria were inflammatory arthritis, malignancy, renal failure, or anaemia.

The clinical examination of patients combined with a knee or hip plain radiograph set the diagnosis of OA. The Kellgren and Lawrence scale [26] was used and patients with grade 3 or 4 OA were scheduled for joint replacement. Age, gender, BMI, and co-morbidities were also recorded.

Blood samples were taken from the patients at their pre-admission visit by a resident orthopaedic surgeon, a week before the operation. The serum levels of 25-hydroxyvitamin D were measured by the Department of Nuclear Medicine, using radioimmunoassay (RIA) (radioactive material supplied by DiaSorin Inc., USA). The patients were categorized into three groups according to their vitamin D status. Vitamin D deficiency was defined as a 25-hydroxyvitamin D level below 20 ng/ml (50 nmol/L) and vitamin D insufficiency as a 25-hydroxyvitamin D level of 21–29 ng/ml (52.5–72.5 nmol/L) [30].

Clinical measurements were recorded by the biochemical laboratory of the Biopathology Department in order to exclude other bone disorders or systemic diseases. The following normal ranges were used: hematocrit (men, normal range: 40–54 %; women, normal range: 37–47 %), haemoglobin (men, normal range: 13.0–18.8 g/dL; women, normal range: 11.6–16.4g/dL), C-reactive protein (normal range: 0.07–8.2 mg/L), urea-BUN (normal range: 9–20 mg/dL), serum creatinine (men, normal range: 0.2-0.6 mg/dL; women, normal range: 0.6–1.0 mg/dL), serum glucose (normal range: 17–43 mg/dL), serum calcium (normal range: 8.1–10.4 mg/dL), and serum phosphorus (normal range: 2.5–4.5 mg/dL).

## Results

In this study, 164 patients were included, 42 (25.6 %) of whom were men and 122 (74.3 %) were women. Age range was 48–86 years [mean = 68.9, standard deviation (SD) = 7.7 years]. All patients were Caucasian; 128 (78 %) of them suffered from knee OA and 36 (22 %) from hip OA. 19.5 % of patients belonged to the Muslim minority.

The levels of vitamin D ranged from 1.61 to 52.19 ng/ml (mean = 13.4, SD = 7.8 ng/ml). It is noteworthy that most patients were vitamin D deficient (81.7 %). 15.2 % of patients were vitamin D insufficient (hypovitaminosis). Only 3 % of patients were vitamin D sufficient (Table 1). Regarding BMI, 6.1 % of patients had optimal weight (BMI range 22–24.9 kg/m^2^), 36.6 % were overweight (BMI range 25–29.9 kg/m^2^) and more than half of the patients (56.7 %) were obese (BMI over 30 kg/m^2^) (Table 1). Additionally, 12 patients, all postmenopausal women, were under medication for osteoporosis with calcium and vitamin D supplements; 7 of them were vitamin D deficient, 4 were vitamin D insufficient and only 1 was vitamin D sufficient.

**Table 1:** Patient groups according to gender, condition, BMI, and vitamin D serum levels

	Number	Percent
Gender		
Female	122	74.4
Male	42	25.6
Condition		
OA knee	128	78.0
OA hip	36	22.0
ΒΜΙ groups		
<22 kg/m^2^	1	0.6
22–24.9 kg/m^2^	10	61
25–29.9 kg/m^2^	60	36.6
>30 kg/m^2^	93	56.7
Vitamin D groups		
Deficiency	134	81.7
Insufficiency	25	15.2
Normal	5	3.0
Total	164	100.0

**Fig. 1:**
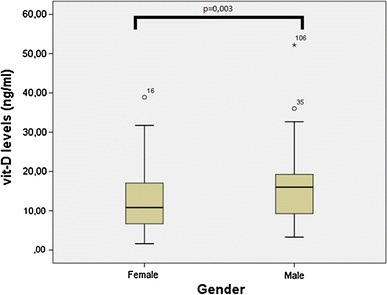
Correlation of vitamin D serum levels and gender

A linear regression model was used to assess links between vitamin D levels and age, gender, and BMI. The regression equation was: VitD levels = 15.238 – (0.226 × BMI) + (0.005 × age) + (3.952 × gender), with *r*^2^ = 0.074 and *p* = 0.006. Male gender had both the highest statistical significance (*p* = 0.004) and impact on the model (*β* = 3.952) in contrast to age (*p* = 0.951, *β* = 0.005). Male gender correlated positively with vitamin D serum levels (*p* = 0.004) (Fig. 1). ΒΜΙ was borderline statistically insignificant (*p* = 0.061) and correlated negatively with vitamin D levels (*β* = −0.226).

Further analysis of vitamin D levels between men and women showed that, on average, men had levels higher by 4.12 ng/ml than women (*p* = 0.003). All analyses were undertaken using the statistical package SPSS for Windows (version 19.0; SPSS Inc., Chicago, USA).

## Discussion

The most important finding of this study is the high prevalence (96.9 % deficiency and insufficiency) of low serum levels of 25-hydroxyvitamin D in a population with OA, in a sunny region of a Mediterranean country. Our study showed that over 4 out of 5 patients with knee or hip OA were vitamin D deficient with serum levels below 20 ng/ml. Several studies have shown a high incidence of vitamin D deficiency in patients with OA of hip or knee [17–19, 23, 24]. Considering the commonness of sunshine in Greece and in relation with existing studies [1–3], we had expected a higher vitamin D status in Greek patients with knee or hip OA. Moreover, the prevalence of vitamin D deficiency in patients with OA scheduled for total hip or knee replacement in our study was higher than the reported values of studies carried out in northern European countries such as Finland [17], Germany [18], and the UK [19] where annual insolation is significantly lower. However, in these countries consumption of vitamin D enriched foods is very common.

We also tried to correlate serum levels of vitamin D with related anthropometric predisposing factors such as age, gender, and BMI. A significant association with gender was observed, with female patients having lower serum levels of vitamin D. A higher prevalence of severe deficiency of vitamin D has also been demonstrated among US adult women compared to men [30]. In a study that took place in Quebec, Canada, gender was not associated with 25(OH)D concentration [31]. In addition, the same study showed that age and BMI were not correlated with 25(OH)D deficiency. This result corresponds to our findings regarding age and BMI. However, considering that BMI was a borderline insignificant predictor of vitamin D levels in our sample, it may be possible that other anthropometric obesity measurements may have stronger association with vitamin D levels. Such measurements could include waist-to-hip ratio and waist-to-height ratio. The notion of association of obesity with low vitamin D levels is supported by Lagunova et al. [32], who found that the prevalence of vitamin D deficiency is dependent on BMI and age separately. The results of that study suggested that 1 in 3 women and 1 in 2 men with BMI ≥40 kg/m^2^ are vitamin D deficient.

The limitations of this study include a small sample size, particularly patients with hip OA. Another limitation is the absence of a control group and the scarcity of available data concerning the vitamin D status in the general population in our region. Despite the aforementioned limitations, the high prevalence of vitamin D deficiency in patients with knee or hip OA scheduled for joint arthroplasty is alarming.
